# Bioactive Molecules, Ethnomedicinal Uses, Toxicology, and Pharmacology of *Peltophorum africanum* Sond (Fabaceae): Systematic Review

**DOI:** 10.3390/plants14020239

**Published:** 2025-01-16

**Authors:** Nkoana I. Mongalo, Maropeng V. Raletsena

**Affiliations:** College of Agriculture and Environmental Science (CAES), University of South Africa, Priva Bag X06, Florida 0710, South Africa; raletmv@unisa.ac.za

**Keywords:** *Peltophorum africanum* Sond, sexually transmitted infections, ethnoveterinary, phytochemistry, antimicrobial, cytotoxicity, in vitro, in vivo, ethnomedicine

## Abstract

Plants have long been used to treat serious illnesses in both humans and animals. A significant underappreciated medicinal tree, *Peltophorum africanum* Sond is utilized by many different ethnic groups to cure a wide range of illnesses. A variety of electronic databases, including ScienceDirect, Scopus, Scielo, Scifinder, PubMed, Web of Science, Medline, and Google Scholar, were used to search the literature on *P. africanum*, using key words such as uses, survey, pharmacology, antigonococcal, toxicity, phytochemistry and others. Further data was obtained from several scholarly theses, dissertations, and books on general plant sciences, ethnomedicine, and other pertinent ethnobotanical topics. The plant species possess very important pharmacological activities in vitro, which includes antimicrobial, anti-HIV, antioxidant, anticancer, antidiabetic, and other activities. Phytochemically, the plant possesses various classes of compounds, dominated by flavonols, which may well explain its wider range of pharmacological activities. Although the plant is promising anti-HIV activity, the mode of action and safety profiles of the plant also need to be explored as its extracts exerted some degree of mutagenicity. It is also important to further explore its ethnoveterinary use against a plethora of nematodes that infects both wild and domestic animals. Given its potent pharmacological activity, the further in vivo studies need to be explored to ascertain the comprehensive toxicology of the plant species, thereby developing possible medications. The plant species may further be elevated to a potent pharmaceutical product against plethora of infections.

## 1. Introduction

Due to inadequate infrastructure, many African communities rely on traditional medicine as their first line of defence. A concerning rise in antimicrobial resistance is exacerbated by opportunistic infections resulting from an increase in HIV-AIDS infections and numerous other illnesses that pose a threat to global health care systems and may necessitate costly medications, even though certain regions of the African continent may have highly relevant and functional infrastructures [1,2,3,4]. These diseases, which can include different types of inflammation and skin malignancies, may be caused by reactive oxygen species and several other factors. They can also lead to debilitating health issues that can impair movement and cause death [5]. While some writers advocate using nanoparticles in combination with medical plant extracts and/or chemicals, several authors suggest using antibiotics in conjunction with medicinal plants [6,7,8,9]. Numerous harmful viruses, fungi, parasites, and pandemics, such as the most recent COVID-19, may cause further ailments.

The genus *Peltophorum* belongs to the family Fabaceae and comprises approximately about 105 plant species which are mostly trees and shrubs distributed worldwide [10,11]. Many members of the genus are used for medicinal purposes hence reported to possess a noteworthy antimicrobial activity [12,13,14,15]. Elsewhere, some important pharmacological activities that includes antimalarial, anticancer, antioxidant, hepatoprotective, analgesic and anti-inflammatory, antiemetic, and antidiabetic activities have also been reported [16,17,18,19,20,21]. Phytochemically, the members of the genus contain a plethora of triterpenes, uncommon amino acids and several phenolic acids which uniquely render the plant species important in traditional medicine [22,23,24,25]. The ethnomedicinal uses, toxicology, phytochemistry of the plant species is well documented. Earlier, some authors reported some scanty versions of such works, as mini reviews, almost a decade ago [26,27]. In the current paper, we are collating such important aspects of the plant species, as a systematic review, as there were other important reports afterwards which puts the plant species in the best position to be used as a pharmaceutical product against a plethora of infections, including extremely resistant microbes. The multipurpose and underrated plant species is used in the treatment of many infections in many southern African countries and has been incorporated in some over-the-counter products sold in south Africa to alleviate many infections that may lead to both mortality and morbidity. Furthermore, the plant species attracted many researchers and hence well studied in the past decade.

### 1.1. Taxonomy of Peltophorum africanum

**Figure 1:**
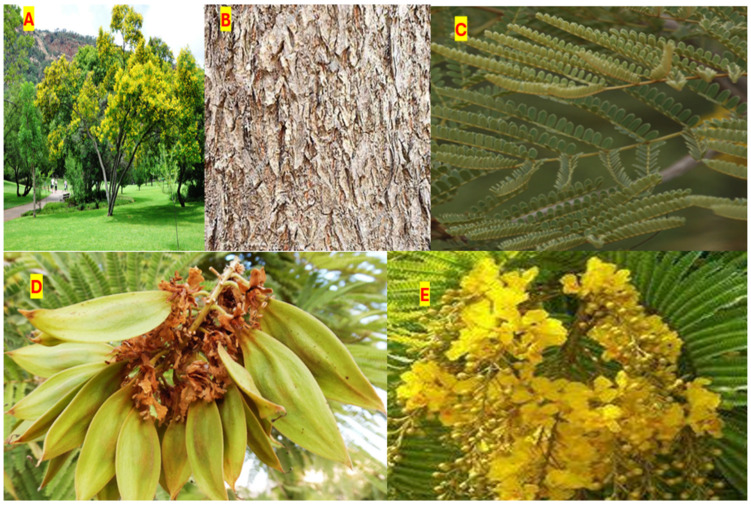
Various parts of *Peltophorum africanum*. Full tree (**A**), Stem bark (**B**), Leaves (**C**), Seeds (**D**), and Flowers (**E**). Pictures of the plant specimen was taken by Dr NI Mongalo, at Edenvale golf Course in Johannesburg.

*Peltophum africanum* Sond (Figure 1A) is a semi-deciduous to deciduous tree of about 15 m in height, with a spreading, untidy canopy. The stem bark in older trees is mostly grooved, rough, longitudinally fissured, and greyish brown in colour (Figure 1B), while the leaves are greenish, alternate; up to nine pairs of pinnae each, compound with a terminal leaf (Figure 1C). Its seeds are embedded with the bean-like structure (Figure 1D), while the flowers, which blooms well in October, form upright, clusters of thin bright yellow with crinkled petals on the ends of branches (Figure 1E).

### 1.2. Distribution and Ecology of the Plant Species

The native range of the plant species ranges from Democratic Republic of Congo and extends Southwards to South Africa. The plant species is mainly Widespread in tropical terrestrial ecosystems with savanna and grasslands as its major habitats [26]. In South Africa the plant species is widespread in the northern provinces, that includes Limpopo, Mpumalanga and northern KwaZulu-Natal. The plant species grows well in well drained soils, mostly in the bushveld and woodlands, on rocky outcrops in sandy soils on riverbanks.

## 2. Ethnomedicinal Uses of *P. africanum*

The ethnomedicinal uses of *Peltophorum africanum* Sond are reported in Table 1. The use of the plant species in South Africa is frequently reported in many ethnic groups in treating various diverse life-threatening illnesses such as sexually transmitted infections, impotence, opportunistic infections associated with HIV-AIDS [27,28,29,30,31,32,33,34,35,36,37,38,39,40,41,42,43,44,45,46,47,48,49,50,51,52,53,54,55,56,57]. Various plant parts may also be used in the treatment and management of various forms of pain and inflammation [52,53,54,55,56,57,58,59,60,61,62,63,64,65,66,67,68,69,70,71,72,73,74,75], various stomach disorders [69,70,71] and gynecological complaints [76,77,78,79,80,81,82,83]. Moreover, there are also reports on the use of the plant species in treating various respiratory infections which may include various strains of virulent corona virus (COVID-19) which was a worldwide pandemic in recent years [84,85,86,87,88,89,90]. Additionally, there are supporting literature sources regarding the use of plant species in different countries to treat respiratory, inflammatory, and ethnoveterinary illnesses as well as other STDs; for these reasons, the current work discusses these topics. These is prevalent in countries such as South Africa, Zimbabwe and Botswana.

**Figure 2:**
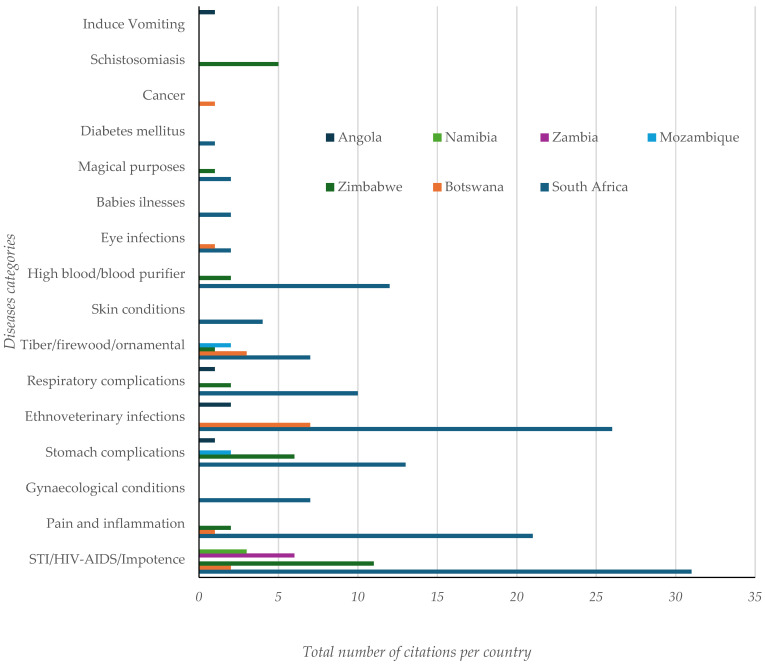
Comparison of the uses of *P. africanum* Sond disease categories and citations per country.

The literature reports on the use of *Peltophorum africanum*, appears in seven different countries in Africa, are mostly reported in South Africa (69.28%), Botswana (7.84%), Zimbabwe (11.11%) and Zambia (3.92%) as shown below (Figure 2). The highest number of citations appears to be in the treatment of sexually transmitted infections, HIV-AIDS and impotence.

Many writers have suggested that the numerous applications of the plant species in KwaZulu Natal, Mpumalanga, and Limpopo, South Africa, may be explained by the fact that these provinces are home to numerous ethnic groups [70,71,74,76,81,91,103,121,123]. It has been suggested by several writers that various ethnic groups living in a similar civilization might employ similar plant species to treat various illnesses [103,121,125]. It’s crucial to remember that, in many cases, a medicinal plant of this kind can be used in conjunction with other plants to increase its biological activity and lessen the possibility of toxicity in both people and animals [27,45]. However, scientific evidence is still lagging on this matter.

## 3. Important Pharmacological Relevance of *Peltophorum africanum* Sond

### 3.1. Antimicrobial Activity

Numerous scholarly sources have documented the plant species’ antibiotic action against a variety of microbes that includes *Enterococcus faecium*, *Staphylococcus aureus*, *Klebsiella pneumoniae*, *Pseudomonas aeruginosa*, and *Enterobacter* spp. (ESKAPE pathogens), rather than the agents that cause STIs itself [44,47,48,49,52,53]. These pathogens have been reported to be highly resistant to many antibiotics used in developing countries worldwide, thereby posing a serious threat to human life and may well cause gastrointestinal, pulmonary and other infections [179,180]. However, some of these pathogens have isolated from many patients infected with urinary tract infections which may be sexually transmissible [181]. Massive CD4+ T lymphocyte depletion is a hallmark of HIV-AIDS development, leading to a variety of diseases, primarily candidiasis and cryptococcosis, which are caused by different fungal strains of *Candida* and *Cryptococcus* respectively [182].

In the current work, antimicrobial activity against STIs, respiratory, skin, and gastrointestinal infections is discussed as shown below (Table 2). Some authors reported the antimicrobial activity of the plant species using both disc diffusion and agar well plate method [47,48,183,184]. These methods are not reliable as the results differ from one laboratory to the other due to factors such as diffusion rate of the extracts, type of agar used and concentration of the bacterial or fungal inoculum. [44,46]. However, it may be used as a starting point in comparing the antibacterial activity of various plant extracts. Many authors reported the antimicrobial activity of plant extracts using broth dilution assay as results may well be compared easily [44,48,50,51,52,53,54] According to Raletsena and Mongalo [5], there is no endpoint in the antimicrobial testing of medicinal plants. However, many other authors recommend the potent minimum inhibitory concentration to be at 0.1 mg/mL, and worthy of further isolation and characterization of the antimicrobial compounds [7,44,45,46,47,48]. Judging by this standard, various extracts from *P. africanum* exhibited a noteworthy antimicrobial activity against a plethora of pathogenic microorganisms which includes *Staphylococcus aureus*, *Ureaplasma urealyticum*, *Trichophyton rubrum*, *Microsporum canis*, *Cryptococcus neoformans*, *Candida albicans*, *Klebsiella pneumoniae*, and *Pseudomonas aeruginosa* [47,48,50,51,128,185]. *C. albicans* is a harmless commensal which may also be implicated as causative agent of oral candidiasis in immunocompromised patients and cohabiting with many other STIs such as syphilis, vaginosis and gonorrhoea [186,187,188]. Although the extracts from the plant species exhibited moderate inhibition of *N. gonorhoea*, *Gardnerella vaginalis* and *Oligella ureolytica* it inhibited *Ureaplasma urealyticum* which causes various genital infections prevalent in pregnant women [189]. According to Mongalo and Mafoko [190], the use of *P. africanum* in treating STIs is in conjunction with other plant species. Many other authors corroborate the idea [44,45,46]. This may well increase the bioactivity against a plethora of pathogens, a synergistic effect. *K. pneumoniae*, and *P. aeruginosa* are implicated in many infections, including urinary tract infections and various gastrointestinal conditions [191,192]. In recent years, *S. aureus*, *T. rubrum*, *M. canis* have been isolated from skin lesions in both human and animals [193]. Although the in vitro antimicrobial activity of *P. africanum* validates the use of the plant species in the treatment of various infections, there is a need to explore the in vivo tests to ascertain its ethnopharmacological relevance. However, it is important to note that although the plant species is reported to treat various pulmonary infections, its biological activity against *Mycobacterium* species is not documented. Furthermore, there is a need to explore the antimicrobial activity of the plant species seasonally and using different collection sites. Some authors reported the varying concentrations of specific compounds in plants, up regulation and down regulation, resulting in different antimicrobial activity [194]. This may well assist traditional healers with the perfect collection time for use in other seasons. However, may plant users are negligible to the idea as the plant is present in all seasons.

When comparing the medicinal plant extract with the positive controls used, particularly in the microplate assay, the controls exhibited better inhibition of the microbes yielding the lowest MIC values. However, it is interesting that in some studies, the medicinal plant extracts exhibited a potent antimicrobial activity relative to the control drugs, particularly against the multi-resistant pathogens isolated from various sources [128,185,196,198,199]. MRSA is an important pathogen posing a serious threat to human life. Furthermore, the bioactivity of the plant species against skin related pathogens such as *T. rubrum, C. neoformans* and *M. canis,* the stomach ulcer implicated *H. pylori and C. albicans* which is a causative agent of multiple opportunistic infections associated with HIV-AIDS validates the use of the plant species in the treatment of such infections. This data may well suggest that the plant species may well be used as an antibiotic to alleviate the *antibiotic* resistance associated with many health care facilities worldwide.

Besides the potent antimicrobial activity, extracts and compounds from *P. africanum* pos sess several other important biological activities as summarized (Supplementary File S1).

### 3.2. Ant-HIV Activity

Acquired immune deficiency syndrome, a chronic illness with possible life-threatening consequences, is brought on by the human immunodeficiency virus (AIDS). This virus primarily targets the immune system, causing the body to become insensitive to outside stimulants. A plethora of African medicinal plants have been evaluated for anti-HIV activity with the intention of managing the infection as it is incurable [204,205]. However, some authors propose that the consumption of these natural health products (NHPs) are not in line with world health organization (WHO) and could not be consumed in conjunction with antiretroviral drugs (ARVs) [206]. Since many NHPs are complex mixtures, it’s likely that they contain organic molecules that have the potential to stimulate or inhibit drug transporters and/or metabolizing enzymes. *Peltophorum africanum* exhibited potent anti-HIV activity in vitro [47,48,57]. The ethanol and aqueous extracts from the stem bark exhibited a noteworthy inhibition of HIV-1 yielding IC_50_ values of 0.05 and 0.10 mg/mL respectively [47,48]. Elsewhere, betulinic acid isolated from the methanolic extract of the stem bark exhibited IC_50_ values of 0.04 and 0.000002 mg/mL against HIV-1-_NL4-3_ and HIV-1-_JRCSF_ respectively [57]. Although in vitro studies may not translate into in vivo studies due to various, the most promising molecules for the continued development of novel antiviral medications turned out to be pentacyclic triterpenes [207,208]. Additionally, substituents attached to betulinic acid’s C-3 and C-28 positions (3β)-3-hydroxy-lup-20(29)-en-28-oic acid) and its analogues were essential for creating novel compounds with enhanced anti-HIV activity [209]. According to Laila et al. [210], triterpenes possess the capacity to halt the HIV life cycle and function as immunomodulators to strengthen the immune systems of those who are infected without causing any known negative consequences. Although botulin possesses potent anti-HIV-1 activity, toxicological aspects and clinical trials needs to be explored. Triterpenes are a class of terpenes composed of six isoprene units with the molecular formula C_30_H_48_ and known to possess antiviral activity against many human infecting viruses such as HSV-1, HIV and other viruses [7]. Triterpenes are divided into two groups, such as tetracyclic that includes dammarane, cucurbitane, lanosterane and cycloartane types, and pentacyclic triterpenes such as oleanane, ursane, lupane, friedelane, hopane and taxaxastane types [211].

### 3.3. Anti-Inflammatory Activity

Numerous chronic illnesses, such as diabetes, intestinal and cardiovascular disorders, various STIs, cancer, and arthritis, are frequently accompanied by inflammation. Recently, an inventory of South African medicinal plants used to treat pain and inflammation have been documented [49,58,59,64,65,75]. The anti-inflammatory activity of *Peltophorum africanum* is well documented in the literature [58,59,60,61,62,63,64,65,66,67,68,69,70,71,72,73,74,75]. The 70% acetone extracts of *P. africanum* leaves exhibited potent anti-inflammatory activity yielding IC_50_ value of 12.42 µg/mL against soybean 15-lipoxygenase (15-LOX) enzyme in vitro compared to other plants extracts [62]. These results were more comparable to the control drug (quercetin) which exhibited IC_50_ value of 8.75 μg/mL. Furthermore, a semi-purified fraction from the leaf’s hexane extracts exhibited an IC_50_ of 0.009 µg/mL against 15-LOX [61]. It is important to note that 15-LOX is an enzyme involved in the metabolism of linoleic acid to leukotriene derivatives. Elsewhere, the fractions and glutinol (pentacyclic triterpene) isolated from leaves hexane extract were reported against cyclooxygenase-1 and 2 (COX1 and COX-2) and nitric oxide synthases [63]. Fraction F3.3 exhibited the IC_50_ value of 0.67 and 0.70 µg/mL against COX-2 and COX-1 respectively, while glutanol exhibited IC_50_ value of 1.22 against COX-1. These results suggest that neither fractions nor glutanol selectively inhibit COX-2. According to some authors, a good anti-inflammatory molecule should inhibit both 15-LOX and COX-2 [212,213]. This is because COX-1 is associated with the normal functioning of the essential body organs. Judging by these standards, the anti-inflammatory activity of *P. africanum* remains questionable and inconclusive. However, it should be noted that traditional medicine use water to extract phytocompounds for the treatment of various infections. Probably, aqueous solution may extract various compounds which may synergistically possess anti-inflammatory activity. On the other hand, glutanol and two fractions F3.0 and F3.3 F3.3 significantly inhibited NO production in a dose-dependent manner [63]. The acetone extract from the leaves exhibited the %NO inhibition of 91.33 at a concentration of 6.25 μg/mL [62]. It is important to note that NO production and COX-2 are implicated in the pathogenesis of inflammation [214,215]. According to Adebayo et al. [60], the mechanism of action of some compounds and fractions from *P. africanum* is through the inhibition of pro-inflammation cytokines such as IL-1β and TNF-α. The bioactivity of the extracts, fractions and glutanol may well serve as anti-inflammatory agents. However, there is still a need to explore the in vivo activities.

### 3.4. Antioxidant Activity

Reactive oxygen species (ROS) and reactive nitrogen species (RNS), which include nitrous oxide, superoxides, hydroxyl radicals, and peroxides, are produced during cellular metabolism in living organisms and are known to be important in oxidative cellular damage and other stresses that lead to a range of diseases that are harmful to the health of humans and animals. Antioxidants are molecules that counteract the very unstable free radicals which are capable of damaging DNA, cell membranes and some other parts of the cell. This may well result in many forms of illnesses that include various chronic infections, including cardiovascular and neurodegenerative infections [216]. According to Scopus citations, the use of 2,2-Diphenyl-1-picrylhydrazyl (DPPH) and 2,2′-azino-bis-3-ethylbenzothiazoline-6-sulfonic acid (ABTS) free radicals is most common in assessing the antioxidant activity of both medicinal plants and fruits [217]. Antioxidant activity of *P. africanum* Sond is well documented [26,27,41,44,62,117,218,219,220]. The methanol and acetone extracts from the leaves exhibited IC_50_ values of 19.0 and 12.50 µg/mL against DPPH respectively [218]. Elsewhere, the methanol extracts from the roots revealed IC_50_ value of 2.24 µg/mL against a similar free radical [41]. The stem bark also exhibited IC_50_ value of 50.0 and 26.4 µg/mL against DPPH and ABTS respectively [44]. Both the acetone extracts from stem bark and the roots exhibited Trolox (TEAC) values of 1.08 and 1.28, suggesting that their antioxidant activity is bigger than that of Trolox [117,219]. Contrarily, the leaves exerted a lower TEAC value of 0.57. The acetone extracts of the leaves further exhibited IC_50_ values of 4.67 and 7.71 µg/mL against DPPH and ABTS respectively and 437.54 μgFe (II)/g in the ferric reducing ability of plasma (FRAP) assay [62]. The ethyl acetate extract from the bark further exhibited IC_50_ values of 3.83, 204.55 and 131.16 µg/mL in a reducing power (Fe^2+^), nitric oxide (NO) and Hydrogen peroxide (H_2_O_2_) assays respectively [220,221]. Besides differences in localities, the differences in antioxidant activity may well be attributed to the age of the plant, the concentration of the free radicals and the choice of the solvent used during extraction. An effective antioxidant ought to exhibit significant efficacy across a minimum of three distinct assays [194]. The roots, stem bark and leaves from *P. africanum* exerted a noteworthy antioxidant activity in DPPH, ABTS, Fe^2+^, NO, H_2_O_2_, FRAP and Trolox assays, rendering it a perfect antioxidant. However, there is still a need to explore the in vivo studies.

### 3.5. Anticancer Activity

A broad range of illnesses shared by the development of uncontrolled malignant tumours and neoplasms are collectively referred to as cancer and can affect any bodily part. According to WHO, cancer is the world’s greatest cause of mortality, accounting for about 10 million fatalities in 2020 [222]. The most common types of cancer are breast, lung, colon and rectum and prostate. The ethyl acetate extract from the stem bark exhibited LC_50_ values of 82.6, 140.09 and 121.07 µg/mL against Human Chang liver cell line at 24, 48 and 72 h incubation period respectively [195]. In the SRB assay, the methanol extract from the leaves exhibited LC_50_ value of >1000 µg/mL against Caco-2 [185]. After 24 h of treatment, the human breast (MCF-7), colon (HT-29), and cervical (HeLa) viable cell counts were lowered by the stem bark ethyl acetate extract to 48.38, 62.36, and 76.10%, respectively, at a concentration of 25 µg/mL [221,223]. It is important to note that these results could not be compared to those of the other studies as the LC_50_ values were not recorded. However, contrast to the negative control, which continued to appear flat and firmly attached to the substrate, the altered control shrank with membrane blebbing, lost its flattened morphology, and split apoptotic bodies because of cytoplasm retraction around the nucleus. This was prevalent in the MCF-7 cell line than the other two cells. According to Shrihastini et al. [224], the plant based anticancer product needs to yield LC_50_ value of ≤30 µg/mL which is also in line with the American Association for Cancer Research. Judging by these standards, the extracts from *P. africanum* Sond exhibited poor anticancer activity against the tested cell lines. However, there is still a need to explore its anticancer activity against a plethora of other cancerous cell lines.

### 3.6. Anti-Diabetic Activity

Diabetes mellitus, which can be divided into Type-1 and Type-2, is a major worldwide health concern that calls for creative solutions to enhance patient outcomes. The heterogeneous nature of the disease has been demonstrated to be a limitation of conventional one-size-fits-all treatment options [225]. Globally, diabetes cases are predicted to rise most in Africa. By 2045, there will likely be 55 million cases of the illness worldwide, a 134% increase from 2021 [226]. The extracts from the leaves immersed in acetone exhibited a noteworthy inhibition of yeast α-glucosidase yielding IC_50_ value of 40 µg/mL compared to acarbose, a control drug which yielded IC_50_ value of 1500 µg/mL [227]. Elsewhere, Zeid et al. [228], reported the antidiabetic effects of ethanol, petroleum ether (PEE), chloroform (CHCl_3_), methanol (AME) and ethyl acetate (EAE) extracts from the leaves. At a concentration of 1000 µg/mL, the EAE, PEE and TEE exhibited α-amylase inhibition of 71.23%, 56.43% and 46.57% respectively in the in vivo studies [228]. At a dose of 300 mg/kg for four consecutive weeks, EAE and TEE reduced serum glucose level (SGL) to 48.3% and 43.9% respectively, compared to that of diabetic control group. These results are not so important because the IC_50_ of the extracts were not reported. However, one can deduce that the extracts from the plant species exhibit moderate to poor inhibition of α-amylase. A potent inhibitor of α-amylase which could be further investigated for the in vivo hypoglycemic effects need to score IC_50_ value of 10.0 µg/mL [229]. It is important to note that the hyperglycemic effect of the plant species is not well studied. Therefore, there is still a need to explore both the in vitro and in vivo antidiabetic effects.

### 3.7. Anthelmintic Effect (Ethnoveteriary)

The anthelmintic effect of the plant species has been evaluated against a plethora of parasites infecting both wild and domestic animals [110,117,118,119,230]. The aqueous extract from both leaves and stem bark combined exhibited a lethal concentration (LD) value of 4.3 and 0.5 mg/mL against *Hymenolepis diminuta* at 1 and 24 h incubation period respectively [230]. The extract further yielded no effect on growth of *Schistosoma mansoni* yielding LD_50_ value of 100 mg/mL. Elsewhere, acetone extracts of the root bark was not found effective against both *Haemonchus contortus* and *Trichostrongylus colubriformis* in sheep at three different doses of 50, 500 and 750 mg/kg [117,118,119]. Furthermore, acetone extracts from leaves, stem bark and roots exhibited an LD_50_ values of 0.62, 0.83 and 0.280 mg/mL in in egg hatch (EH) study against *T*. *colubriformis* respectively. The extracts further exhibited LD_50_ values of 0.72, 0.37 and 0.28 mg/mL against a similar parasite in the larval development (LD) study respectively. In both LD and EH studies, the LD_50_ of the roots were highly comparable to the positive control, Thiabendazole. Although acetone was used as an extracting solvent, the use of the plant species in the treatment of various anthelmintic infections is promising. Elsewhere, the hot and cold-water extracts from the stem bark exhibited 58.0 and 55.33% inhibition of *H. contortus* in an EH assay [110]. The exracts further exhibited 100% inhibition of a similar parasite in the LD assay. However, there is a need to isolate and characterize the active phytocompounds and further explore their mode of action, especially as *P. africanum*’s bioactivity is most prominent against *H. contortus* and *T. colubriformis.*

## 4. Toxicology

The toxicological aspects of extracts from *P. africanum* Sond are summarized as shown below (Table 3). The methods used includes brine shrimp lethality assay (BSLA), in vitro cytotoxicity against a plethora of normal human and animal cell lines, and antimutagenicity against *Salmonella typhimurium* TA98 and *Salmonella typhimurium* TA100 with and without S9 metabolic activation. Histological parameters on the In vivo studies of rats treated with extracts from *P. africanum* are also reported.

### 4.1. Brine Shrimp Lethality Assay (BSLA)

The acetone extracts from roots, stem bark and leaves did not show toxicity in the brine shrimp assay, each yielding LD_50_ > 1000 μg/mL against *Artemia nauplii* [117]. In other cases, after 24 and 48 h of incubation, the methanol extracts from stem bark at a dose of 2 mg/mL produced 20.40 and 25.85% inhibition (mortality rate), respectively [66,231]. Recently, the organic and aqueous extracts from stem bark and roots were evaluated for toxicity against *Artemia franciscana* at 24 h incubation period [232]. At 0.94 mg/mL, the organic (1:1 methanol: dichloromethane) extract from the leaves exerted some degree of toxicity, yielding a 53% mortality rate against the *Artemia* spp. Contrarily, the aqueous extracts from roots and leaves exhibited 49.0 and 47.0% inhibition respectively at a concentration of 1 mg/mL. The organic extracts’ toxicology was also examined in combination with the extracts from two other medicinal plants such as *Bridelia cathartica* G. Bertol., and *Rhoicissus digitata* (L.f.). At a concentration of 0.67 mg/mL, the results showed that the mixture was non-toxic. Although the extracts from *P. africanum* Sond are generally non-toxic to various *Artemia* species in the BSL assay, it is important to note that the results of the BSL assay do not translate into the mammalian cell studies [194]. Additionally, the toxicity of the solvent, rather than the drug or extract itself, causes the drug carrier, which is frequently a solvent utilized primarily for extracting the solvent, to provide false-positive results [233].

**Table 3:** Toxicology of extracts from *P. africanum* Sond.

Method Used	Extracts/Solvents	Results Obtained	References
Brine shrimp lethality assay (BSLA)	Roots, stem bark and leaves extracted with acetone.	The extracts exhibited LD_50_ > 1000 μg/mL in the BSLA assay against *Artemia nauplii*	[117]
	Stem bark extracted with methanol.	At a concentration of 2 mg/mL, the extract exhibited a mortality rate of 20.40 and 25.85% against *A. nauplii* after 24 and 48 h of incubation respectively.	[66,231]
	Stem bark extracted with methanol	The extract exhibited LD_50_ value of 882.0 μg/mL against *A. nauplii.*	[197]
	Stem bark extracted with water and 1:1 methanol: dichloromethane	The organic extract at 0.91 mg/mL exhibited 53% mortality rate against the *Artemia* spp. 24 h incubation period	[232]
Cytotoxicity	Roots extracted with ethanol	The extract yielded LD_50_ value of 133.3 µg/mL against Vero cells.	[41]
	Leaves extracted with acetone	The extracts exhibited LD_50_ value of 103.45 µg/mL against Vero cells.	[62]
	Leaves extracted with 70% acetone	The extract exhibited LD_50_ value of 400 µg/mL.	[85,234]
	Stem bark extracted with water and ethanol	At 100 µg/mL, the aqueous and organic extracts from the stem bark exhibited 98 and 99% cell growth inhibition against HKE cells while the aqueous extracts from the stem bark revealed LD_50_ value of 1073 µg/mL against HDF cells	[51,117]
	Stem bark extracted with water and ethanol	Ethanol and water extracts exhibited LD_50_ values of 0.72 and 1.07 mg/mL RAW 264.7 murine macrophage and human dermal fibroblasts respectively	[66,67]
	Leaves extracted with acetone	The extract exerted LD_50_ value of 669.20 µg/mL against THP-1 macrophages and did not show any sign of toxicity against the human lymphatic endothelial cell	[85,235]
Antimutagenicity	Stembark extracted with water, ethanol, petroleum ether and dichloromethane	The aqueous extracts exhibited the highest His+ revertant/plate of 48.5 at a concentration of 50 µg/mL in an assay with metabolic activation	[47,49]
	Leaves and roots extracted with water and 1:1 methanol: dichloromethane	Organic extract from the roots exhibited average revertant colonies equal to 1142 against *Salnonella typhimurium* TA100, while the aqueous extract from leaves exhibited 2462 colonies against *Salnonella typhimurium* TA98.	[232]
Histopathologic effects	Leaves extracted with ethanol	The extracts showed significant histological alterations characterized by vacuolation of islets of Langerhans cells.	[228]

### 4.2. Cytotoxicity

The cytotoxicity of extracts from *P. africanum* Sond has been well studied against Vero Monkey kidney cell line [41,62,85,117,234]. The ethanol extract from the roots exhibited LC_50_ value of 133.3 µg/mL against the Vero cells, while acetone extract of the leaves exhibited LD_50_ value of 103.45 µg/mL against similar cell line [41,62]. Elsewhere, the acetone leaves extracts exhibited LD_50_ value of 515.60 µg/mL, while 70% acetone extracts exhibited LD_50_ value of 400 µg/mL [85,234]. Contrarily, the acetone extracts from the roots, stem bark and leaves exhibited LC_50_ value of ›1000 µg/mL against similar cell line. The significant difference in terms of LD_50_ may well be attributed to the quantities and growth stage of the seeded cells, age of the plant specimen, environmental conditions, location and season of collection. Other authors investigated the cytotoxicity of the extracts from *P. africanum* against human dermal fibroblast (HDF), human lymphatic endothelial (HLE) and human embryonic kidney epithelial (HEKE) cell lines in vitro [51,66,67]. At a concentration of 100 µg/mL, the aqueous and organic extracts from the stem bark exhibited 98 and 99% cell growth against HKE cells while the aqueous extracts from the stem bark revealed LD_50_ value of 1073 µg/mL against HDF cells [51,117]. Elsewhere, the acetone extracts from the leaves exerted LD_50_ value of 669.20 µg/mL against THP-1 macrophages and did not show any sign of toxicity against the human lymphatic endothelial cell [85,235]. It is important to note that THP-1 macrophages are responsible for the modulation of the monocytes and macrophages activity, while may well serve as a promising cellular model to study biological mechanisms of major depression and antidepressant drug response, hence important in pharmaceutics [236]. According to Shrihastini et al., plant based phytocompounds and extracts are toxic when yielding LD_50_ values of ≤30 µg/mL against normal human cell lines [224]. Judging by this standard, extracts from *P. africanum* are non-toxic to several cell lines, rendering the extracts safe for consumption. However, caution still needs to be exercised as consumption may well be dependent on human body weight and quantities consumed. According to Mboweni, extracts from *P. africanum* are non-toxic even when combined with other medicinal plants [235]. It is also important to note that the plant species is used in conjunction with other medicinal plants as an immune booster “Amandla” which also treats erectile dysfunction [234]. Such immune booster has also been evaluated against Vero cells and was found to be non-toxic. In the quest to find new antimicrobial agents, plant species with no toxicity against normal cell lines are relevant. Higher LD_50_ and lower MIC favors higher selectivity index (SI) which is indicative of higher safety margin [7,211].

### 4.3. Antimutagenicity

Recently, the antimutagenic effects of both roots and leaves (aqueous and organic) extracts has been evaluated against both *Salmonella typhimurium* TA98 and *Salmonella typhimurium* TA100 with and without S9 metabolic activation [47,49,232]. It is important to note that Ames test without S9 metabolic activation detects direct mutagens while the Ames test with S9 metabolic activation allows the detection of indirect mutagens. The petroleum ether, dichloromethane, ethanol and aqueous extracts from *P. africanum* Sond stem bark exhibited varying degrees of His+ revertant/plate. The aqueous extracts exhibited the highest His+ revertant/plate of 48.5 at a concentration of 50 µg/mL in an assay with metabolic activation [47,49]. Although all the extracts were tested at three different concentrations (50, 500 and 5000 µg/mL), all the samples did not yield a dose dependent increase in colonies, hence classified as non-mutagenic towards *S. typhimurium* TA98. Contrarily, both the 1:1 methanol: dichloromethane extracts from leaves and roots, at a concentration of 5 mg/mL, exerted some degree of mutagenicity against *S. typhimurium* TA98 and TA100 yielding 2462 and 1142 Average revertant colonies respectively [232]. The mutagenicity of the roots extracts was almost two-fold higher than that of a control drug which yielded 624 average revertant colonies, while that of the leaves was extremely higher, almost four-fold compared to the control drug. However, it should be noted that the aqueous extracts of both the leaves and the roots were non mutagenic to both *S. typhimurium* TA98 and TA100 at a similar concentration. It is not worrying that organic extracts revealed mutagenicity. In traditional medicine, aqueous extracts are used to treat various devastating human and animal infections, hence safe for consumption with no mutagenicity.

### 4.4. Histopathologic Effects

The histopathology of rats treated orally with *P. africanum* Sond leaf ethanol extracts was investigated [228]. The diabetic rats administered with the extracts showed significant histological alterations characterized by vacuolation of islets of Langerhans cells. Research in this area still lags and needs to be further explored.

## 5. Phytochemistry of *P. africanum*

Using qualitative phytochemical analysis, various parts of *P. africanum* Sond exhibited a desirable quantity of tannins, alkaloids, flavonoids, steroids and terpenoids and some varying degrees of total phenolic contents (TPC) and total flavonoid contents (TFC) [44,62,85,110,199,234]. Elsewhere, various compounds were detected using gas-chromatography mass spectrometry (GC-MS) [228]. The nature or type of the compounds characterized names of the compounds and methods of characterization are shown below (Table 4).

**Figure 3:**
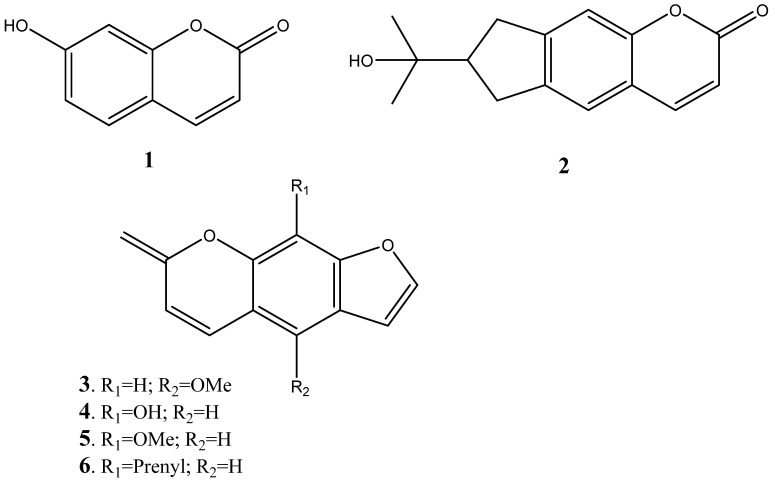
Coumarins isolated from leaves of *Peltophorum africanum*.

The 90% ethanolic extract of the leaves yielded compounds such as 7-hydroxycoumarin (**1**), marmesin (**2**), bergamptin (**3**), xanthonin (**4**), xanthotoxol (**5**), and imperatonin (**6**) as shown in Figure 3 [237].

It is important to note that 7-hydroxycoumarin is pharmaceutically relevant as it possesses relevant biological activity along with its derivatives. The biological actions of 7-hydroxycoumarin, including its anti-inflammatory, antioxidant, neuroprotective, antipsychotic, antiepileptic, antidiabetic, antibacterial, antiviral, and antiproliferative properties, make it a promising candidate for therapeutic uses [238,239]. To create 7-hydroxycoumarin-based metal complexes with enhanced pharmacological action, 7-hydroxycoumarin ligands have also been used. In addition to its use in medicine, 7-hydroxycoumarin analogues have been developed as fluorescent probes to identify biologically significant species like enzymes, lysosomes, and endosomes, or to track the actions of cells and proteins, as well as the progression of different illnesses [240].

**Figure 4:**
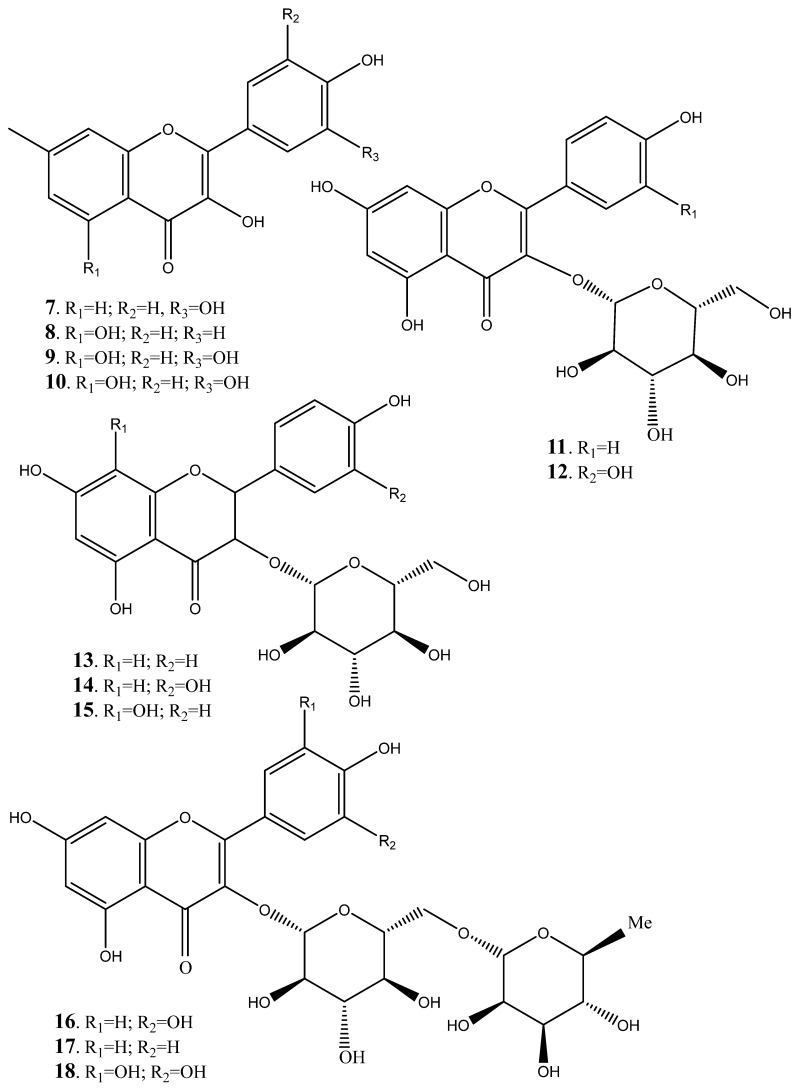

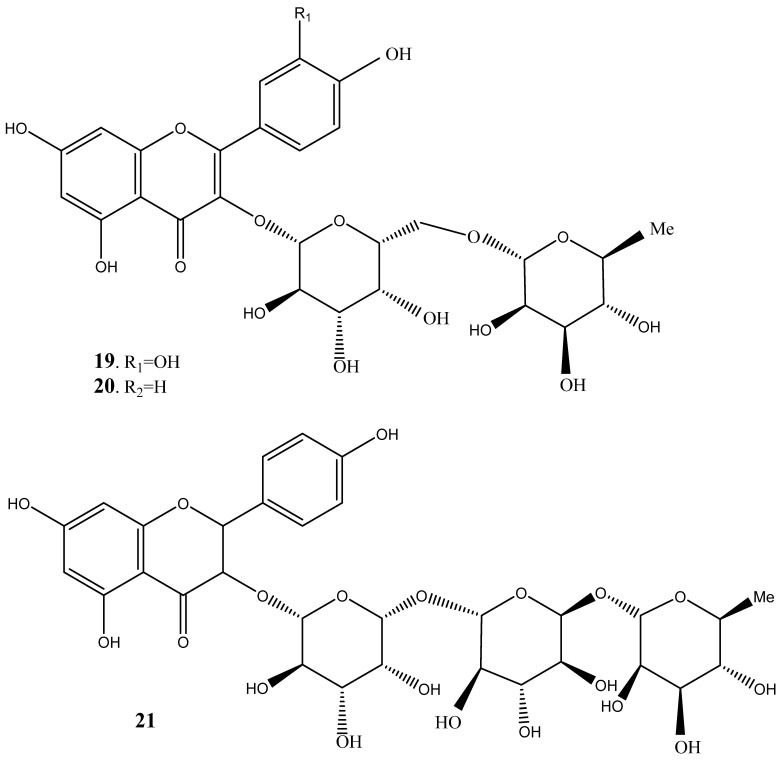

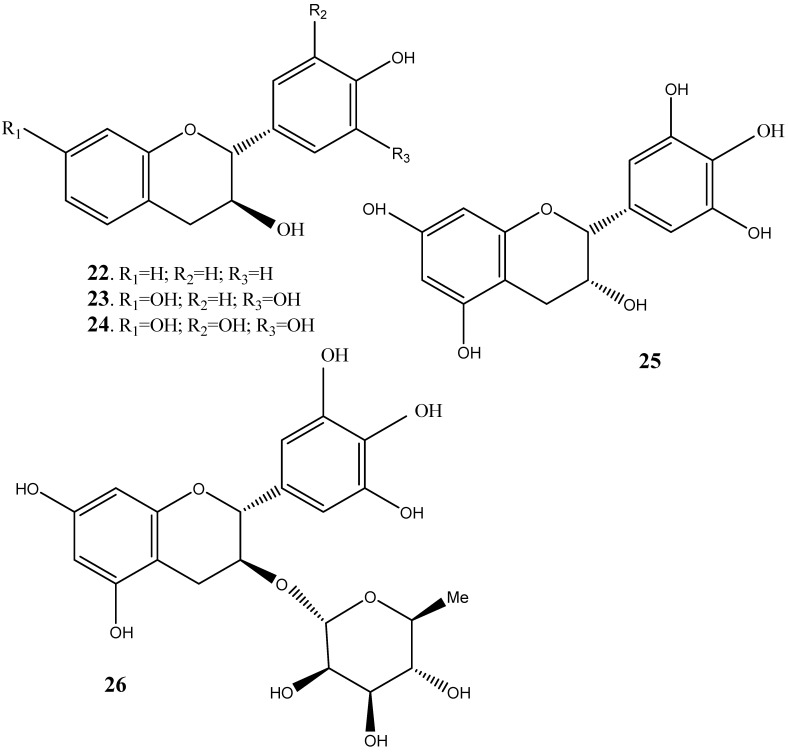
Various flavanols isolated and characterized from various parts of *Peltophorum africanum*.

Various flavanols (Figure 4) have also been isolated from heartwood, stem bark and the flowers [241,242,243,244,245,246,247]. Flavonols such as fisetin **(7**), kaempferol (**8**), myricetin (**9**), quercetin (**10**), astralagin (**11**), isoquercitrin (**12**), kaempferol-3-galactoside (**13**), quercetin-3-galactoside (**14**), herbacetin-3-galactoside (**15**), rutin (**16**), nicotiflorin (**17**), myrcetin-3-rutinoside (**18**), quercetin-3-rhamnosylgalactoside (**19**), kaempferol-3-rhamnosylgalactoside (**20**) and kaempferol-3-rhamnosylglucosylgalactoside (**21**) have been identified from heart wood, stem bark and flowers [241,242]. Elsewhere, flavan-3-ol (**22**), fisetinidol (**23**), robinetinidol (**24**), catechin (**25**), and catechin3-o-rhamnoside (**26**) has been characterised from heart wood, stem bark, flowers and leaves [243,244].

Recently, positive antimicrobial, antiviral, anti-inflammatory, anti-allergenic, and anti-cancer properties have been widely reported from catechin, quercetin and rutin [248,249,250]. Catechins improve the usefulness of healthy functional meals and biocosmetics by facilitating their increased absorption and penetration into the skin and body and reportedly for human consumption and used as a strong and stable antioxidant, hence alleviating the devastating impact free radicals such as ROS and RNS in human body [248]. Quercetin and rutin offers the protective effects of essential human organs such as liver, kidneys, and heart by counteracting organ toxicity [249,250,251,252].

**Table 4:** Classes of compounds characterized from *P. africanum* Sond.

Class of Compound	Compound Number	Name of Compound	Method of Characterization	Reference(s)
Coumarins	**1**	7-hydroxycoumarin	HR-UPLC/PDA/ESI/MS Analysis	[237]
	**2**	Marmesin	HR-UPLC/PDA/ESI/MS Analysis	[237]
	**3**	Bergamptin	HR-UPLC/PDA/ESI/MS Analysis	[237]
	**4**	Xanthonin	HR-UPLC/PDA/ESI/MS Analysis	[237]
	**5**	Xanthotoxol	HR-UPLC/PDA/ESI/MS Analysis	[237]
	**6**	Imperatonin	HR-UPLC/PDA/ESI/MS Analysis	[237]
Flavanols	**7**	Fisetin		[243,244]
	**8**	Kaempferol	^13^C NMR	[241,242]
	**9**	Myricetin	^13^C NMR	[241,242]
	**10**	Quercetin	^13^C NMR	[241,242]
	**11**	Astralagin	^13^C NMR	[241,242]
	**12**	Isoquercitrin	^13^C NMR	[241,242]
	**13**	Kaempferol-3-galactoside	^13^C NMR	[241,242]
	**14**	Quercetin-3-galactoside	^13^C NMR	[241,242]
	**15**	herbacetin-3-galactoside	^13^C NMR	[241,242]
	**16**	rutin	^13^C NMR	[241,242]
	**17**	Nicotiflorin	^13^C NMR	[241,242]
	**18**	Myrcetin-3-rutinoside	^13^C NMR	[241,242]
	**19**	quercetin-3-rhamnosylgalactoside	^13^C NMR	[241,242]
	**20**	Kaempferol-3-rhamnosylgalactoside	^13^C NMR	[241,242]
	**21**	Kaempferol-3-rhamnosylglucosylgalactoside	^13^C NMR	[241,242]
	**22**	flavan-3-ol	^1^H NMR and CD data	[243,244]
	**23**	Fisetinidol	^1^H NMR and CD data	[243,244]
	**24**	Robinetinidol	^1^H NMR and CD data	[243,244]
	**25**	Catechin	^1^H NMR and CD data	[57,243,244]
	**26**	Catechin3-*O*-rhamnoside	^1^H NMR and CD data	[57,243,244]
Triterpenes	**27**	betulinic acid	^13^C NMR and ^1^H NMR	[57,237,242]
	**28**	β-amyrin	^13^C NMR and ^1^H NMR	[57,237,242]
	**29**	β-sitosterol	^13^C NMR and ^1^H NMR	[57,237,242]
	**30**	Stigmasterol	^13^C NMR and ^1^H NMR	[57,237,242]
Benzoids	**31**	Berginin	^13^C NMR and ^1^H NMR	[249,250,251,252]
	**32**	Norbegenin	^13^C NMR and ^1^H NMR	[249,250,251,252]
	**33**	11-O-(E)-p-coumaroylbergin	^13^C NMR and ^1^H NMR	[249,250,251,252]
	**34**	11-O-galloylbegenin	^13^C NMR and ^1^H NMR	[249,250,251,252]
	**35**	Gallic acid	^13^C NMR and ^1^H NMR	[249,250,251,252]
	**36**	methylgallate	^13^C NMR and ^1^H NMR	[249,250,251,252]
	**37**	Methyl gallic acid	^13^C NMR and ^1^H NMR	[249,250,251,252]
	**38**	chlorogenic acid	^13^C NMR and ^1^H NMR	[249,250,251,252]
Lactones	**39**	2-(3,4-dihydroxyphenyl (	^13^C NMR and ^1^H NMR	[249,250,251,252]
	**40**	2-3,4,5-trihydroxyphenyl	^13^C NMR and ^1^H NMR	[249,250,251,252]
Condensed Flavanoids	**41**	Bissextol	^13^C NMR and ^1^H NMR	[249,250,251,252]
	**42**	Cyanomaclurin	^13^C NMR and ^1^H NMR	[249,250,251,252]
	**43**	Cyanomaclurin analog	^13^C NMR and ^1^H NMR	[249,250,251,252]

**Figure 5:**
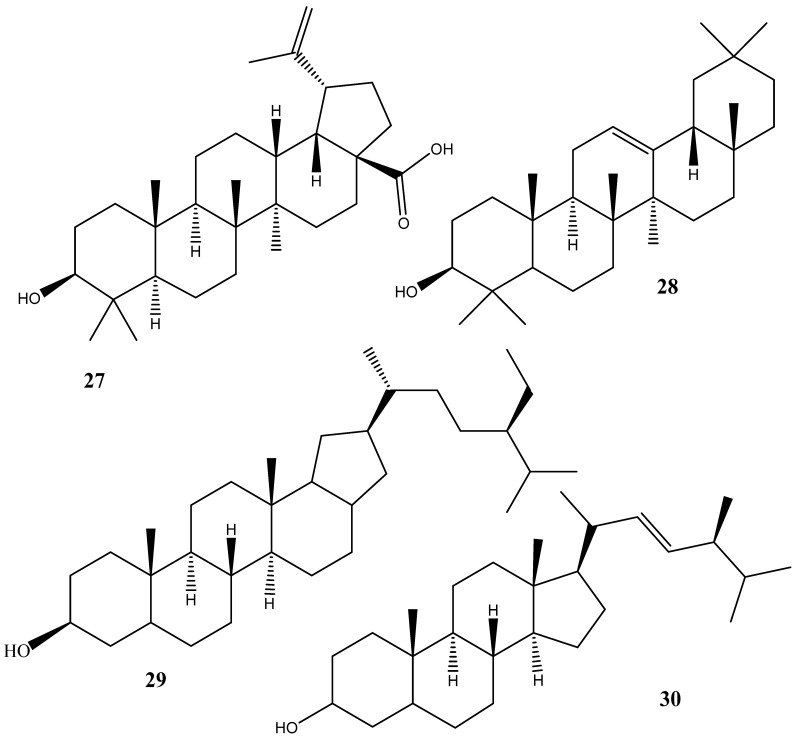
Some triterpenes isolated and characterized from various parts from *Peltophorum africanum*.

Some pharmacologically important terpenoids have been identified from both the leaves and stem bark [57,237,242]. Compounds such as betulinic acid (**27**) along with its analogs, β-amyrin (**28**), β-sitosterol (**29**), and stigmasterol (**30**) have been identified from various leaves and stem bark extracts (Figure 5). Its anti-HIV activity has also been documented [57]. Along with many of its analogs and derivatives, BA as a multitarget compound offers defense against oxidative stress, inflammation, cancer, diabetes, liver, and cardiovascular disorders. It fights tumor cells by causing apoptosis.

**Figure 6:**
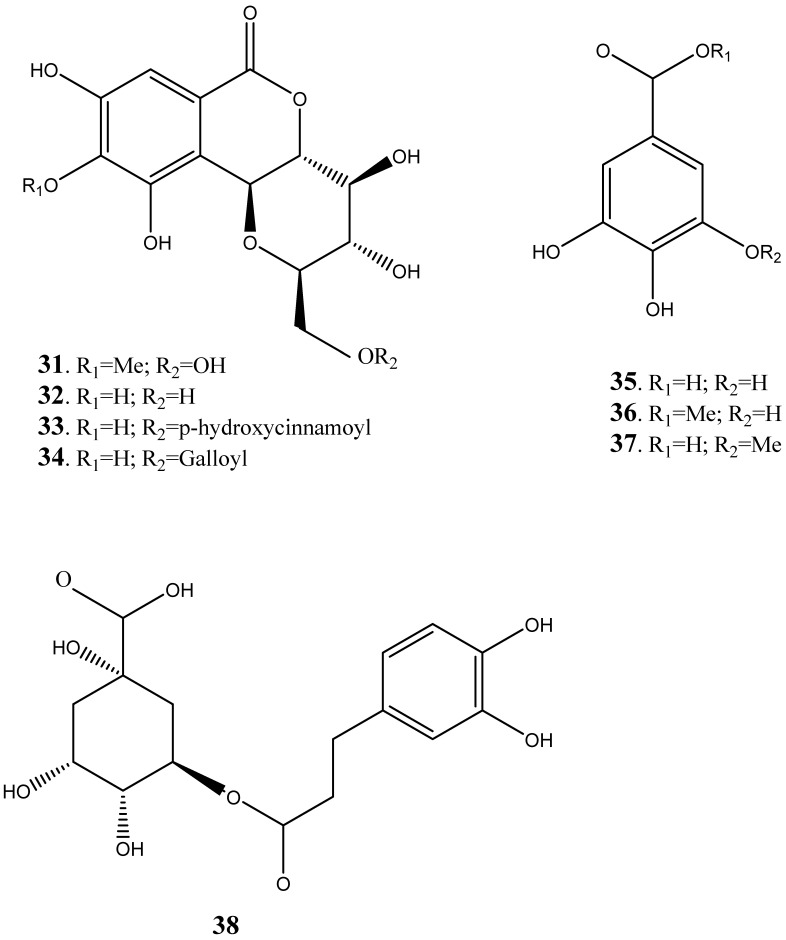
Benzoids isolated and characterized from various parts from *Peltophorum africanum*.

**Figure 7:**
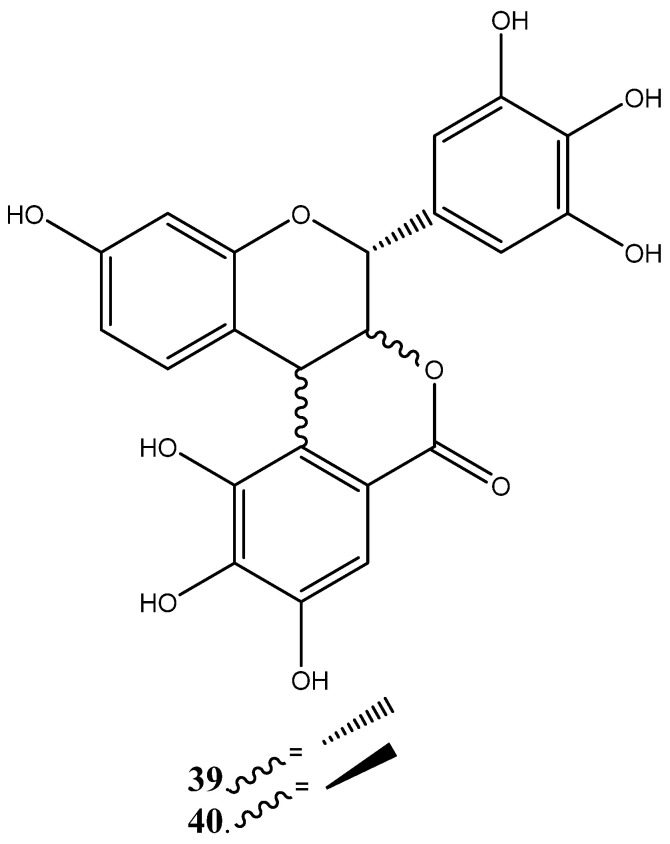
Lactones isolated and characterized from heartwood of *Peltophorum africanum*.

**Figure 8:**
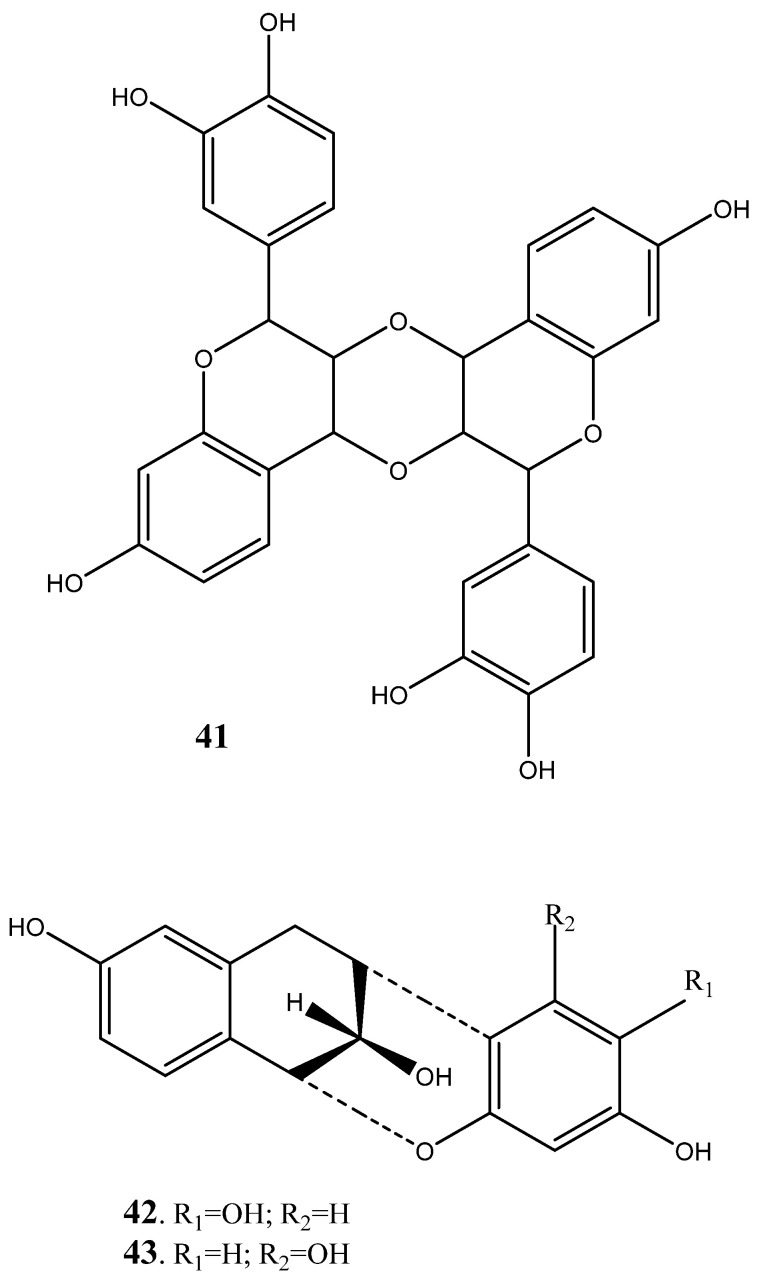
Condensed flavonoids isolated and characterized from heartwood of *Peltophorum africanum*.

The other groups of compounds identified from the plant species include benzoids (Figure 6), lactones (Figure 7), and condensed flavonoids (Figure 8). The compounds such as berginin (**31**), norbegenin (**32**), 11-O-(E)-p-coumaroylbergin (**33**), 11-O-galloylbegenin (**34**), gallic acid (**35**), methyl gallate (**36**), 3-O-Methyl gallic acid (**37**) and chlorogenic acid (**38**) were identified from the heartwood, roots and stem bark [249,250,251,252]. Furthermore, two lactones such as 2-(3,4-dihydroxyphenyl (**39**) and 2-3,4,5-trihydroxyphenyl (**40**) lactones have also been identified from the heart wood, while condensed flavonoids such as bissextol (**41**), cyanomaclurin (**42**) and cyanomaclurin analog (**43**) have also been identified from the heartwood.

Several benzoids, condensed flavonoids, and lactones from the plant species exhibited notable antimicrobial activity [241,244]. However, the mode of action of such compounds remains unknown. It is also important to note that benzoids are versatile in nature and many pharmacologically important compounds may well be synthesised from their rings, resulting in many anticancer, anti-inflammatory, anthelmintic and antimicrobial agents [253,254]. In conclusion, the variety of phytocompounds from the plant species may well explain the important pharmacological activities reported in the current work. However, in many instances, the mode of action remains unexplored. It is also these pharmacological activities which resulted in some over the counter products possessing *P. africanum* as one of the ingredients [46,66,145]. However, it is important to also further explore the toxicological aspects of such products.

## 6. Materials and Methods

### 6.1. Strategy for Literature Search

The information reported in the current paper was collected from a literature search using various computerized databases such as ScienceDirect, Scopus, Scielo, Medline, Scifinder, Web of Science, and Google Scholar. Additional information was further retrieved from various academic dissertations, theses and general plant sciences, ethnomedicine, and other relevant ethnobotanical books. This was conducted following the guidelines by the Preferred Reporting Items for Systematic Reviews and Meta-Analyses (PRISMA) statement [255]. Keywords such as Africa, *Peltophorum africanum* Sond, toxicology, cytotoxicity, ethnomedicinal uses, survey, phytochemistry, antioxidant, antiparasitic, anthelmintic, anti-inflammatory, anticancer, antidiabetic, and ethnopharmacological aspects were used interchangeably.

### 6.2. Data Mining to Generate the Inventory/Data

**Figure 9:**
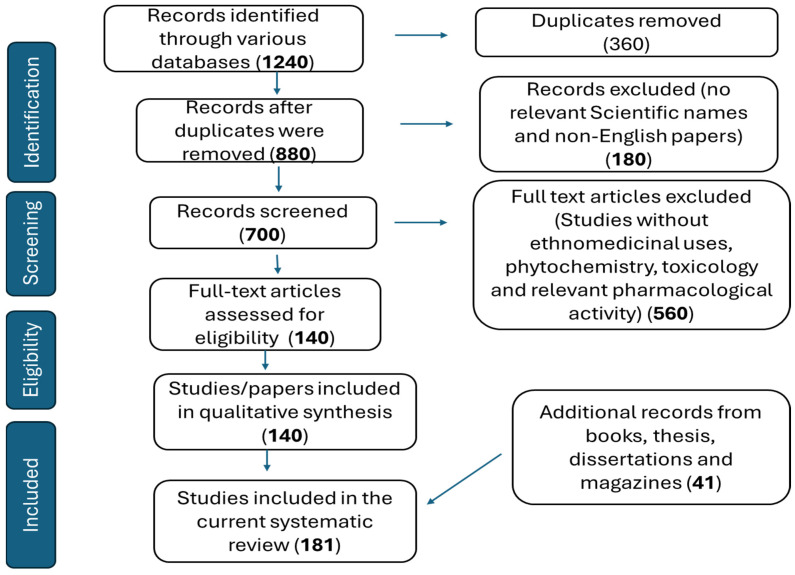
Various sources extracted from the literature used in the current systematic review.

The inclusion criteria were the following: (1) the literature source has ethnobotanical or ethnopharmacological context, and articles should be ethnobotanical field studies/surveys reporting on plant(s) with an indication as used for treating a specific infection, both human and animals and other relevant uses; (2) the study location must be Africa; (3) study must focus on *Peltophorum africanum* Sond; (4) study must be written in English. On the other hand, the exclusion criteria were the following: (1) articles with no scientific plant names; (2) Abstracts and papers with no relevance to pharmacological activity, uses, phytochemistry and toxicological aspects. Data were collected with help from library staff at the University of South Africa (Florida Campus). In the search engine, plant species with only the genus name, *Peltophorum*, were omitted in the current work. The papers, books, and other sources used in the current work were screened for inclusion. Papers appearing as duplicates, cited in an ambiguous abstract form, or not in English were excluded (Figure 9). The task was conducted by the first author and confirmed by the second author. From each of the relevant articles, plant parts, methods of preparation, and use in the management and treatment of several human and animal infections were recorded. Furthermore, papers with specific pharmacological activity, phytochemistry, and toxicology were grouped. The data in this systematic review dates from 1978 to 2014.

## 7. Conclusions

Although the study is limited by the lack of the pharmacological activities of the phytocompounds isolated and characterized from the plant species and the mode of action of some extracts and compounds, there is an enormous information from the literature supporting the use of *Peltophorum africanum* Sond in treating various infections, hence possessing antimicrobial activity against a plethora of pathogenic strains in vitro, particularly against the most pathogenic strains yielding MIC values of ≤1 mg/mL. Toxicologically, the extracts and the compounds from the plant species exhibited some mutagenicity, although not toxic to several normal human cell lines. Various extracts from the plant species further exhibited noteworthy anthelmintic activity in both egg hatch and larval development experiments in vivo, supporting the use of the plant species in the management of several ethnoveterinary infections. In the antimicrobial, antidiabetic, anti-inflammatoory, antioxidant, anti-HIV activity the extracts and compounds from the plant species exhibited potent activity in vitro with IC_50_ better than the control drugs used. These results may well validate the use of the plant species in the treatment of such infections, including gastrointestinal, skin, boosting the immune system in patients with immunocompromised patients, delaying the progression of HIV-AIDS fever, several forms of diabetes, pain and inflammation. However, there is still a need to explore the in vivo studies. The biological activities observed may well be attributed to the variety of compounds embedded within various parts of the plant species, mostly synergistically. Although benzoids, triterpenes are ported from the plant species, flavonoids remain the dominant compounds.

## Figures and Tables

**Table 1:** Ethnomedicinal uses of *Peltophorum africanum* Sond.

Country (Indigenous Names)	Plant Part Used	Ethnomedicinal Uses	Possible Mode of Administration	References
South Africa (African Blackwood, African Wattle, African-wattle, Mosêtlha (Musese, Natal Wattle, Rhodesian Black Wattle, Rhodesian Wattle Rooikiaathout (Umthobo)	Stem bark	Various types of sexually transmitted infections, impotence, and various opportunistic infections associated with HIV-AIDS.	The decoction is drunk until the infection subsides. The pounded stem bark is licked to treat impotence.	[27,28,29,30,31,32,33,34,35,36,37,38,39,40,41,42,43,44,45,46,47,48,49,50,51,52,53,54,55,56,57]
	Whole plant	Whole plant treats pain and inflammation in the entire body (painful body and tooth, joints, backache, pains after giving birth).	The various parts are either immersed in water t equal proportions or boiled to be drunk twice a day if the infection is severe or once if not.	[28,52,53,58,59,60,61,62,63,64,65,66,67,68,69,70,71,72,73,74,75]
	Whole plant	Gynecological conditions including infertility and menorrhagia.	The plant is boiled, and the infusion is taken orally to treat infections. For infertility, the stem bark is mixed with *Cassia abbreviata* Oliv. stem bark and *Solanum aculeastrum* Dunal roots.	[76,77,78,79,80,81,82]
	Roots and stem bark	To treat stomach disorders including diarrhea and “makgoma”.	The bark piece is kept in the mouth like a lozenge to prevent stomach infiltration in children known as “makgoma”. Stem bark may be combined with that of *Cassia abbreviata* Oliv., immersed in water and drunk to treat stomachache. Roots are boiled and drunk orally, 2 tin cups a day.	[28,35,52,53,69,70,71,83,91,92,93,94,95]
	Whole plant	Various ethnoveterinary infections in cattle, goats, fowls and pigeons.	Several pieces are boiled, allowed to cool and	[28,96,97,98,99,100,101,102,103,104,105,106,107,108,109,110,111,112,113,114,115,116,117,118,119,120]
	Roots and stem bark	Are used to treat respiratory infections (cough, sore throat, asthma, colds, fever, and flu related infections including pneumonia and tuberculosis).	The stem bark is pounded and mixed with dried powdered roots of *Acacia erioloba*, *Ximenia caffra* and dried bark of *Sclerocarrya birrea*. Taken orally with warm water to treat asthma. Roots are boiled and drunk thrice a day to treat tuberculosis.	[35,52,53,84,85,86,87,88,89,90]
	Stem bark	Firewood, fence post, carving bowls (timber) and ornamental purposes (shade and decoration of homes)	The wet stem bark is cut and carved with several tools such as sharp axe, knives and stones to the shape of the desired final product. Such products may well generate income.	[35,121,122,123,124,125,126]
	Stem bark	Used to treat skin and related infections, including wounds, sores, and rash.	Stem bark is dried pounded amd applied to sceral wound types and skin infections directly after wetting with water to adhere to the wounds.	[35,111,127,128]
	Stem bark	Treat high blood pressure and blood purifier	The stem bark is immersed in water and the resulting liquid Is drunk thrice a day until the infection subsides.	[90,121,122,123,124,125,126,127,128,129,130,131]
	Stem bark	Eye infections	The stem bark is immersed in water and the resulting liquid is used to wash the infected eyes daily in the morning for a period of two weeks.	[35,52,53,131]
	Stem bark	Babies’ illnesses (“hlogwana”; Metopic sutures)	The head of a new brn is cut a little with a sharp new razor. The pounded stem bark mixed with Vaseline is the applied to the whole head, including the wound created by razor cut.	[35,54]
	Leaves and stem bark	are used for magical purposes		[35,54]
	Leaves and stem bark	Diabetes	The stem bark and leaves are boiled and mixed with cow milk to treat diabetes. The liquid is drunk 4 hourly until the infection subsides.	[72]
	Leaves and stem bark	Madness	Not mentioned	[132]
Botswana (Nzeze, Mosetlha)	Stem bark	Cancer	The stem bark decoction is drunk thrice a day.	[133]
	Stem bark.	To treat various ethnoveterinary infections	Stem bark is mixed with that of *Sclerocarrya birrea* Hochst. boiled and taken orally until the infection heals.	[134,135,136,137,138,139,140]
	Stem bark	Various types of sexually transmitted infections, impotence and various opportunistic infections associated with HIV-AIDS.	For STIs, the stem bark is boiled and taken orally. For impotence, the dried pounded stem bark is licked with a tongue in the evening before bed or added to “mageu” to be drunk by men. It may be mixed with *Securidaca longipedunculata* root bark. In cases of oral candidiasis, the boiled decoction is cooled and then gargled.	[141,142]
	Roots	To treat various types of pains and inflammation	Roots are boiled and taken orally.	[142]
	Roots	Used to treat eye infections	By immersing in water and washing eyes daily in the morning.	[133]
	Stem bark	Firewood, fence post, carving bowls (timber) and ornamental purposes (shade and decoration of homes)	Stems are chopped, allowed to dry and used to make firewood. Bigger stems may also be used as fence posts.	[143,144,145]
Zimbabwe (Muzeze, Dzedze, Mudjiza, Zeze, Musambanyoka, Nyakambariro, Nyamanyoka)	Stem bark and roots	To treat urinary tract infections (schistosomiasis)	Both stem bark and roots are either immersed in water or boiled and resulting liquid is drunk for two weeks.	[146,147,148,149,150]
	Whole plants	Religious ceremonies and rituals (Magical)	The poundd plant material is immersed in water and springled all over the home of a deceased and in ceremonies where religious functions may be celebrated.	[151]
	Stem bark	Blood purification	The infusion of the stem bark with salt is used as a blood purifier	[149,151]
	Roots and stem bark	To treat stomach disorders including diarrhea	Both roots and stem bark are mixed, pounded and immersed in ater. The resulting liquid is drunk twice a day to trert diarrhea.	[149,150,151,152,153,154]
	Whole plants	Sexually transmitted infections and opportunistic infections associated with HIV-AIDS.	The whole plant boiled and taken orally.	[146,149,150,154,155,156,157,158,159,160,161]
	Whole plant	Pain and inflammation (painful body and tooth, joints, backache	The roots are boiled and the resulting liquid is gargled to treat a painful tooth. The stem bark may be pounded and mixed with Vaseline. The resulting product is applied directly to the painful area until the infection subsides.	[149,154]
	Roots	Roots are boiled and taken orally to treat various infections that includes respiratory infections (cough and tuberculosis),	Roots are boiled and taken orally thrice a day for a period of 1 month.	[154,161]
	Whole plant	Firewood, fence post, carving bowls (timber) and ornamental purposes (shade and decoration of homes)	Sharp objects are used to cut the stem into a shape of the desired product. Some plants are grown to provide shade in many towns, cities and rural homes.	[162]
Mozambique (Txuva)	Whole plant planted	Firewood, fence post, carving bowls (timber) and ornamental purposes (shade and decoration of homes)	Sharp objects are used to cut the stem into a shape of the desired product. Some plants are grown to provide shade in many towns, cities and rural homes.	[163,164]
	Roots and stem bark	Used to treat stomach disorders including diarrhea.	Roots and or stem bark are boiled, and the resulting liquid is taken orally until the infection subsides.	[165,166]
Zambia (Muparara, Muzenzenze)	Whole plant	The whole plant is used to treat unidentified sexually transmitted infections and opportunistic infections associated with HIV-AIDS.	whole plant is boiled, and the resulting decoction is drunk daily until the infection subsides.	[160,167,168,169,170]
Namibia	Whole plant	The whole plant is used to treat unidentified sexually transmitted infections and opportunistic infections associated with HIV-AIDS.	Whole plant is boiled, and the resulting decoction is drunk daily until the infection subsides.	[171,172,173]
Angola (Omumpalala, Mupalala, Mupapa, Ompalala, Omupalala)	Whole plant	Used to treat various ethnoveterinary infections	Immersed in water and drunk by fowl. Boiled and taken orally to treat diarrhea in cattle.	[174,175]
	Stem bark and roots	Respiratory infections.	Stem bark and roots are boiled and drunk until the infection subsides.	[176]
Swaziland (Sikhabamkhombo)	Stem bark	Stomach complaints	The stem bark and roots are ground and 30 g is immersed in water. The resulting liquid is drunk to cure various forms of Stomach complaints	[177]
	Stem bark and roots	Induce vomiting	Stem bark and roots are ground, immersed in water and resulting mixture is drunk to induce vomiting	[178]

**Table 2:** Antimicrobial effects of *P. africanum* Sond against some important pathogens.

Plant Parts and Solvent Used	Pathogens Tested	MIC Against Relevant Pathogen	Reference(s)
Roots extracted with 70% ethanol	*Neisseria gonorrhoea, Oligella ureolytica*. *Gardnerella vaginalis* and *Candida albicans*	The extract exhibited MIC value of 1.6 mg/mL against both *N. gonorrhoea* and *O. ureolytica*. Furthermore, the extract exhibited MIC values of 12.5 and 3.1 mg/mL against *G. vaginalis* and *C. albicans* respectively. Ciprofloxacin was used as a positive control and yielded MIC value of 0.01 against *C. albicans* and ≤0.01 against all other pathogens.	[38,39,40,41]
Stem bark extracted with acetone	*Escherichia coli* and *Staphylococcus aureus.*	The extract exhibited MIC values of 2.0 and 0.39 against *E. coli* and *S. aureus* respectively. Streptomycin sulphate was used as a positive control and yielded MIC value of 0.03 mg/mL against both microbes.	[44]
Stem bark extracted with water and petroleum ether (PE)	*Staphylococcus aureus.*	Aqueous extract exhibited MIC value of 0.098 mg/mL against *S. aureus*. PE extract inhibited the growth of *Neisseria gonorrhoea* by 56% in a Disk-diffusion assay. Neomycin was used as a positive control and exhibited MIC value of 0.006 mg/mL.	[47,48]
Roots extracted with water and 1:1 methanol: dichloromethane	*Trichomonas vaginalis, Ureaplasma urealyticum, G. vaginalis, N. gonorrhoea* and *Oligella ureolytica*	The aqueous extract exhibited MIC value of 0.50 mg/mL against *G. vaginalis* and *N. gonorrhoea* while the organic extract exhibited MIC value of 0.04 mg/mL against *U. urealyticum.* Aqueous and organic extracts further exhibited MIC values of 4.0 and 1.0 mg/mL against *C. albicans* and *O. ureolytica* respectively. Furthermore, aqueous and organic extracts exhibited MIC values of >16.0 and 2.0 mg/mL against *T. vaginalis* respectively. Ciprofloxacin was used as a positive control and yielded MIC values of 0.20, 0.63, 0.39, 0.12 and 0.04 µg/mL against *U. urealyticum O. ureolytica, G. vaginalis, Trichomonas vaginalis* and *N. gonorrhoea* respectively.	[50,51]
Leaves extracted with dichloromethane: methanol (1:1)	*Klebsiella pneumoniae, Pseudomonas aeruginosa and Staphylococcus aureus.*	The extract exhibited MIC value of 1.0 against all the selected pathogens. Ciprofloxacin was used as a positive control and yielded MIC values of 0.31 µg/mL against the selected pathogens.	[52,53]
Stem bark extracted with methanol	*Moraxella catarrhalis, K. pneumoniae* and *S. aureus*	The extract exhibited MIC values of 1.0 mg/mL against *Klebsiella pneumoniae* and a further 2.0 mg/mL against both *Moraxella catarrhalis* and *S. aureus*. Ciprofloxacin was used as a positive control and yielded MIC values of 0.05, 0.03 and 0.07 µg/mL against *M. catarrhalis, K. pneumoniae* and *S. aureus* respectively.	[66]
Leaves and roots extracted with dichloromethane and ethanol respectively	*S. aureus*	Both extracts exhibited MIC values of 0.08 mg/mL against *S. aureus*. Gentamycin was used as a positive control and exhibited MIC value of 0.04 mg/mL.	[117]
Stem bark extracted with acetone and hot water.	*Trichophyton rubrum Microsporum canis* and *C. albicans*	Acetone and water extracts exhibited MIC values of 0.08 and 0.02 mg/mL against *C. albicans* respectively after 24 h of incubation. Furthermore, acetone and water extracts exhibited MIC values of 0.04 and 0.02 mg/mL against *T. rubrum* respectively at 24 h incubation period. Acetone and water extracts exhibited MIC values of 0.63 and 1.25 mg/mL against *M. canis* respectively at 24 h incubation period. Amphotericin B was used as a positive control and yielded MIC values of 2.50, against both *C. albicans* and *Microsporum canis* and further MIC value of 0.31 mg/mL against *T. rubrum* at 24 hr incubation period.	[128]
Stem bark extracted with ethyl acetate	*Plesiomonas shigelloides*	The extract exhibited zone of inhibition of 26.5 mm at a concentration of 50 mg/mL and MIC value of 0.156 mg/mL against *P. shigelloides* Tetracycline was used as a positive control and exhibited MIC vale of 10 µg/mL against *P. shigelloides.*	[195]
Stem bark extracted with ethyl acetate	Various *Helicobacter pylori* strains	The extract further exhibited highest zone of inhibition of 20 mm and MIC value of 0.01 mg/mL against various *H. pylori strains.* Metronidazole was used as positive control and exhibited MIC value ranging from 0.01 to ≥6.25 mg/mL.	[196]
Leaves extracted with methanol	Methicillin resistant *Staphylococcus aureus* (MRSA).	The extract exhibited MIC value of 0.0078 mg/mL against the selected pathogen. The microbe was resistant to azithromycin, tetracycline, meropenem, sulfamethoxazole, cefoxitin, amikacin and gentamycin.	[185]
Leaves extracted with methanol	*S. aureus* and *Streptococcus group* G strains	The extract exhibited MIC values of 5.0 and 2.5 mg/mL against *S. aureus and Streptococcus* group G strains respectively. No control drug used.	[197]
Stem bark extracted with methanol	*Shigella sonnei* and *Salmonella typhi.*	The extract exhibited zone of inhibition (ZI)of 10 and 12 mm against *S. sonnei* and *S. typhi.* Oxytetracycline was used as appositive control and exhibited ZI values of 17 and 16 mm against *S. sonnei* and *S. typhi* respectively.	[183]
Leaves extracted with dichloromethane.	*C. albicans* and *Cryptococcus neoformans*	The extract exhibited MIC values of 0.08 and 0.02 mg/mL against *C. albicans* and *C. neoformans* respectively. Amphotericin B was used as positive control and exhibited MIC value of 2.5 mg/mL against the selected microbes both at 24 and 48 h incubation period.	[198,199]
Stem bark extracted with methanol	*Campylobacter jejuni*	The extract suppressed growth of several species of *C. jejuni* yielding 72% inhibition at a concentration of 3 mg/mL. Gentamycin was used as positive control and exhibited 100% inhibition of the microbe at 0.09 mg/mL.	[200]
Roots extracted with methanol	*C. albicans.*	The extract exhibited MIC value of 16 mg/mL against clinical isolates of *C. albicans.* No control drug used.	[201]
Stem bark extracted with acetone	*Bacillus subtilis* and *Pseudomonas aeruginosa*	The extract yielded ZI of 18 mm and 14 mm *P. aeruginosa* and *B. subtilis* respectively. Gentamycin was used as positive control and yielded ZI values of 30 and 19 mm against *B. subtilis* and *P. aeruginosa* respectively.	[184]
Stem bark extracted with methanol	*S. flexineri*	The extract’s MIC value against the microbe was 1.5 mg/mL, while gentamycin’s was 0.008 mg/mL.	[202]
Roots extracted with methanol	*E. coli*, *S. aureus*, *Streptococcus* group G, *Pseudomonas aeruginosa* and *C. albicans*.	The extract exhibited an average MIC value of 10 mg/mL against all the tested microbes. No control drug used.	[203]

## Data Availability

Data of the manuscript is within the paper.

## Supplementary Materials

The following supporting information can be downloaded at: https://www.mdpi.com/article/10.3390/plants14020239/s1. File S1: Other Important biological activities *Peltophorum africanum*.
