# Supplementary material for: Bioactive Molecules, Ethnomedicinal Uses, Toxicology, and Pharmacology of Peltophorum africanum Sond (Fabaceae): Systematic Review
Source: Plants (Basel). 2025 Jan 16;14(2):239. doi: 10.3390/plants14020239 (PMC11768249; doi:10.3390/plants14020239)
Supplement: Supplementary file 1 [file plants-14-00239-s001.zip › plants-3291360-supplementary.pdf]

Other Important biological activities *Peltophorum africanum*.

Activity investigated	Tested material	Activity and results	Experimental evidence assessment	References
**Anti-HIV**	Stem bark extracted with methanol and water	Methanol and water extracts exhibited HIV-1 yielding IC_{50} values of 0.05 and 0.10 mg/ml respectively.	Positive evidence; dose dependence	[47,48].
	Betulinic acid (BA)	BA exhibited IC_{50} values of 0.04 and 0,000002 mg/ml against HIV-1-$_{NL4-3}$ and HIV-1-$_{JRCSF}$ respectively.	Positive evidence; dose dependence	[57].
**Anti-inflammatory**	Semi-purified fraction from hexane leaves extract	The fraction exhibited an IC_{50} of 0.009 µg/ml against soybean 15-lipoxygenase (15-LOX), more active than quercetin, a control drug, which exhibited IC_{50} value of 8.75 µg/ml.	Positive evidence; dose dependence	[61].
	70% acetone extract from leaves	The extract exhibited potent anti-inflammatory activity yielding IC_{50} value of 12.42 µg/ml against 15-LOX enzyme *in vitro* compared to other plants extracts.	Positive evidence; dose dependence	[62].
	Fraction F3.3 and glutanol	The fraction exhibited the IC_{50} value of 0.67 and 0.70 µg/ml against COX-2 and COX-1 respectively, while glutanol exhibited IC_{50} value of 1.22 against COX-1.	Positive evidence; dose dependence	[63].
	Glutanol and fractions F3.0 and F3.3	All the tested plant materials inhibited NO production in a dose-dependent manner	Inconclusive evidence, dose dependence	[63].
**Antioxidant**	Roots extracted with methanol	The extract revealed IC_{50} value of 2.24 µg/ml against DPPH	Positive evidence; dose dependence	[41].
	Stem bark extracted with acetone	The extract revealed IC_{50} value of 50.0 and 26.4 µg/ml against DPPH and ABTS respectively	Positive evidence; dose dependence	[44]
	Leaves extracted with acetone	The extracts exhibited IC_{50} values of 4.67 and 7.71 µg/ml against DPPH and ABTS respectively and 437.54 µgFe (II)/g in the ferric reducing ability of plasma (FRAP) assay	Positive evidence; dose dependence	[62].
	Roots and stem bark extracted with acetone	Both the acetone extracts from stem bark and the roots exhibited Trolox (TEAC) values of 1.08 and 1.28.	Positive evidence; dose dependence	[117].
	Leaves extracted with both methanol and acetone	Methanol and acetone extracts exhibited IC_{50} values of 19.0 and 12.50 µg/ml against DPPH respectively	Positive evidence; dose dependence	[218].
	Stem bark extracted with ethyl acetate	Extract exhibited IC_{50} values of 3.83, 204.55 and 131.16 µg/ml in a reducing power (Fe^{2+}), nitric oxide (NO) and Hydrogen peroxide (H_2O_2) assays respectively	Positive evidence; dose dependence	[220,221].

**Anticancer**	Stem bark extracted with ethyl acetate	The extract from the stem bark exhibited LC_{50} values of 82.6, 140.09 and 121.07 μg/ml against Human Chang liver cell line at 24, 48 and 72 hr incubation period respectively	Positive evidence; dose dependence	[195].
	Leaves extracted with methanol	The extract exhibited LC_{50} value of >1000 μg/ml against Caco-2.	Positive evidence; dose dependence	[185].
	Stem bark extracted with ethyl acetate	After 24 hours of treatment, the extract lowered the viable cell count by the 48.38, 62.36, and 76.10% against the human breast (MCF-7), colon (HT-29), and cervical (HeLa) respectively, at a concentration of 25 μg/ml.	Positive evidence; dose dependence	[221,223].
**Antidiabetic**	Leaves extracted with acetone	The extracts exhibited an inhibition of yeast α-glucosidase yielding IC_{50} value of 40 μg/ml.	Positive evidence; dose dependence	[227].
	Leaves extracted with ethanol, petroleum ether (PEE), chloroform ($CHCl_3$), methanol (AME) and ethyl acetate (EAE)	At a concentration of 1000 μg/ml, the EAE, PEE and TEE exhibited α-amylase inhibition of 71.23%, 56.43% and 46.57% respectively in the *in vivo* studies.	Inconclusive evidence, not dose dependent	[228].
**Anthelmintic effect.**	leaves and stem bark extracted with water	The extracts synergistically exhibited a lethal concentration (LD) value of 4.3 and 0.5 mg/ml against *Hymenolepis diminuta* at 1 and 24 hr incubation period respectively. The extracts yielded LD_{50} value of 100 mg/ml. against *Schistosoma mansoni*.	Inconclusive evidence, dose dependence	[230].
	Root bark extracted with acetone	The extract was found effective against both *Haemonchus contortus* and *Trichostrongylus colubriformis* in sheep at three different doses of 50, 500 and 750 mg/kg.	Inconclusive evidence, not dose dependence	[117–119].
	Leaves, stem bark and roots extracted with acetone	The extracts exhibited an LD_{50} values of 0.62, 0.83 and 0.280 mg/ml in in egg hatch (EH) study against *T. colubriformis respectively.*	Positive evidence; dose dependence	[117–119].
	Leaves, stem bark and roots extracted with acetone	The extracts exhibited LD_{50} values of 0.72, 0.37 and 0.28 mg/ml against a similar parasite in the larval development (LD) study respectively.	Positive evidence; dose dependence	[117–119].
